# In vivo and in vitro biocompatibility study of novel microemulsion hybridized with bovine serum albumin as nanocarrier for drug delivery

**DOI:** 10.1016/j.heliyon.2019.e01858

**Published:** 2019-06-06

**Authors:** Mahmoud Gharbavi, Hamidreza Kheiri Manjili, Jafar Amani, Ali Sharafi, Hossein Danafar

**Affiliations:** a Department of Pharmaceutical Biomaterials, School of Pharmacy, Zanjan University of Medical Sciences, Zanjan, Iran; b Pharmaceutical Nanotechnology Department, School of Pharmacy, Zanjan University of Medical Sciences, Zanjan, Iran; c Zanjan Pharmaceutical Biotechnology Research Center, Zanjan University of Medical Sciences, Zanjan, Iran; d Cancer Gene Therapy Research Center, School of Pharmacy, Zanjan University of Medical Sciences, Zanjan, Iran; e Zanjan Applied Pharmacology Research Center, Zanjan University of Medical Sciences, Zanjan, Iran; f Applied Microbiology Research Center, Systems Biology and Poisonings Institute, Baqiyatallah University of Medical Sciences, Tehran, Iran

**Keywords:** Pharmaceutical chemistry, Bovine serum albumin nanoparticles (BSA NPs), Biocompatibility, Co-delivery, Microemulsion, Multifunctional, T-BSA-ME

## Abstract

The present study aimed to synthesize triacetin-microemulsion (T-ME) and T-ME hybridized with bovine serum albumin nanoparticles (T-BSA-ME) having narrow particle size distribution and versatile carrier systems as a novel microemulsion system. The suggested ME system was characterized by Fourier Transform Infrared spectroscopy (FTIR), Differential Scanning Calorimetry (DSC), and Atomic Force Microscopy (AFM). The physicochemical properties of microemulsion system including particle size, PDI and ζ-potential, refractive index, Conductivity, %Transmittance, pH, and rheological behavior were also evaluated. In vivo biocompatibility was done using Median Lethal Dose (LD 50) calculated and trialed to evaluate the acute toxicity. In Addition, hemolysis and leukocyte proliferation assay were characterized to evaluate in-vitro biocompatibility of the suggested MEs systems. Moreover, cytotoxicity of MEs systems was also investigated on HFF-2 and HEK-293 cells. The presence of BSA NPs as a macromolecular biomaterial hybridized with T-ME reduced the cytotoxicity. The properties of the suggested MEs system proposed the T-ME hybridized with BSA-NPs as a promising candidate for co-delivery and multifunctional biomedicine applications.

## 1 Introduction

Microemulsions have many advantages such as, notably, optical transparency, low viscosity, single optically isotropic and thermodynamically stable isotropic systems, small particle size of <100, and the ability to be formed spontaneously without any need to high-shear equipment [1].

Due to having many unique properties, they are used in several pharmaceutical applications such as parenteral delivery, oral drug delivery, topical drug delivery, ocular and pulmonary delivery and biotechnology [2]. Microemulsions are composed of four components: the surfactant, co-surfactant, oil and aqueous phase [3]. They are divided into mainly three categories: water-in-oil (W/O), bicontinuous, and oil-in-water (O/W) microemulsions. The latest microemulsion type has been used as hydrophobic drug carrier, such as an anticancer drug. To fabricate of microemulsions, triacetin (glycerol triacetate) has been utilized as an oil phase (internal phase) [4]. It has several unique characteristics like nontoxicity, high hidrophobic drug solubilization capacity and Self-Emulsifying formulations ability, so it can be used as a co-solvent as well as an emulsification aid. Polyoxyethylene (20) sorbitan monooleate (Tween 80) and sorbitan monooleate (Span 80) are used as surfactant. Since Tween 80 have bulky polyoxyethylene groups, with a HLB of 15.0 and more solubility in water, it tends to form O/W emulsions [5]. Span 80, with a HLB value of 4.3, is a viscous, lipophilic, emulsifying liquid agent which tends to form W/O emulsions [6]. Moreover, Tween 80 and Span 80 are generally recognized as safe and are approved for use in a number of pharmaceutical, cosmetic, and food products because they are nonirritant and have a low potential for toxicity [6]. The mixture of Tween 80 and Span 80 enhanced the long-term stability as well as inclination to form expanded films in mixed monolayers, leading to an increase in hydrophobic area [7].

Propylene glycol and glycerol have a salting-in effect, making it suitable for being used as a co-surfactant [8, 9]. They are incorporated into the surfactant layer and thus, increases the interfacial fluidity. Furthermore, PG will decrease the polarity of the water because PG is mainly soluble in water, increasing the single-phase area. This phenomenon is very important in terms of application and economy. It should be noted that the main challenging factor in nanomedicine is how NPs can distinguish healthy tissue from damaged ones. For selective targeting of NPs into the pathogen sites, nanoparticle delivery system required targeting. Surface functionalizing NPs is the widely-used technique that allows conjugation with targeting ligands. It is inherently able to direct selected binding to cell types or states and therefore, confer “smartness” to NPs. The NPs surface is stimulated to conjugate with one or multiple cell ligands, resulting in an intelligent status. This technique has been widely used in various contexts. The major disadvantage of microemulsions, especially oil in water (O/W) type, is the lack of suitable surface that can accomplish this technique. To overcome the limitations of microemulsions, microemulsions hybridized with BSA nanoparticle were used as a novel strategy. BSA was widely used because of its non-toxicity, biodegradability, non-immunogenicity, water solubility, availability and its low cost, because large quantities of it can be readily purified from bovine blood [10]. Bovine serum albumin (BSA) is an important mode protein that has a molecular weight of 66 kDa and consists of about 583 amino acids with a structure like heart-shaped globular protein [11]. It has a variety of multiple functional groups at the surface, such as thiol, amine and carboxyl groups. As a result, its surface is activated by any functional groups (thiol, amine and carboxyl groups), as ligand, and can bind to significant amounts of the drug. We also PEGylated BSA NPs, to impart the *in vivo* longevity to drug carrier and reduce the protein binding (opsonization) [11, 12]. In this study, bovine serum albumin nanoparticles (BSA NPs) were used as excellent and powerful macromolecular biomaterials to activate microemulsions surface. In this method, BSA-PEG NPs were utilized as fraction of the microemulsions aqueous phase that activate microemulsions surface and stabilize the suggested microemulsions by the induced negative charge. The suggested microemulsions systems, triacetin in water (O/W) hybridized with BSA NPs (T-BSA-ME), were characterized by FT-IR, transmittance percentage, refractive index, pH analysis, conductivity, and thermal analysis. The cytotoxicity of MEs systems was evaluated as *in vivo* and *in vitro*. *In vitro* cell viability studies of microemulsions systems were carried out using HFF-2 and HEK-293 cells by MTT assay at different microemulsions concentrations for 48 h of incubation. In addition, biocompatibility of MEs systems was carried out using hemolysis and leukocyte proliferation assay.

## 2 Materials and methods

### 2.1 Materials

Triacetin (CAS.102-76-1), polyoxyethylene sorbitan monooleate (Tween 80- CAS.9005-65-6), propylene glycol (CAS.57-55-6), sorbitan monooleate (span80-CAS.1338-43-8)), BSA (CAS.9048-46-8), PEG (Mn = 6000 Da) (CAS. 25322683) and MTT reagent were purchased from Sigma Aldrich Company in Germany. Other chemicals and solvents of high purity were purchased from Emertat chimi (Tehran- Iran).

### 2.2 Synthesis BSA-PEG –NP

BSA-PEG-NP were prepared by desolation technique as reported by Merodio et al [13]. About 200 mg BSA and 20 mg PEG-6000 were dissolved in 3m of deionized water under constant Stirring and then, kept at room temperature for 15 min, followed by the adjustment of pH to 9.0 with 0.2 M NaOH. Then, it was added to the above solution dropwise under constant stirring at room temperature with a constant addition rate of 2 ml/min. In order to stabilize particles, they were cross-linked with 150 μl of 0.8% glutaraldehyde solution under stirring for 24 hours. The obtained NPs were subjected to centrifugation at 10000 rpm for 10 min for 3 cycles and the NPs were redispersed to the original volume in deionized water. The solution was lyophilized using freeze dryer and then, the dried NPs were collected as BSA-PEG NPs.

### 2.3 Synthesis O/W microemulsions hybrid with BSA-PEG NPs

Modified O/W microemulsions were prepared by Tween80 and Span80 as mixture surfactant and propylene glycol as co-surfactant that were hybridized with BSA-PEG NPs. According to optimum formulation, 2.8% (0.28 g) BSA-PEG NPs was added to 9.72 g water; then, 17.48% (2.5g)W/W tween 80 and 2.97% (0.42g)W/W span 80 (as mixed surfactant), 4.37% (0.62g)W/W PG (as co-surfactant) were added to the aqueous solution, respectively, at 35–40 °C under vigorous stirring for 15 min. Then, 5.24% (0.75g) W/W triacetin (as oil phase) was added dropwise to the aqueous phase to form O/W microemulsions hybridized with BSA-PEG NPs.

### 2.4 Physicochemical characterizations

#### 2.4.1 FT-IR characterization of microemulsion hybrid with BSA-PEG NPs

Fourier transform infrared (FT-IR) spectroscopy of BSA, PEG, BSA-PEG NPs, microemulsions free BSA-PEG NPs and microemulsions BSA-PEG NPs were performed on KBr pellets with an FT-IR spectrophotometry (Spectrum Two, USA). The spectra were scanned over the range 400–4000 cm^−1^.

#### 2.4.2 Particle size and ζ-potential analysis of samples perpetrated

Dynamic light scattering (DLS) was used to determine mean particle size and size distribution (polydispersity index) of the modified O/W microemulsions by employing the Zeta sizer Nanoseries instrument (Malvern Nano ZS®, Malvern Instruments Ltd, Worcestershire, UK). 0.5 ml of microemulsions or modified microemulsions was diluted with 1.5 ml of ultra-pure water in a clean Malvern sample vial. The hydrodynamic, polydispersity index (PDI) and ζ-potential of all samples were analyzed by DLS.

#### 2.4.3 Morphology analysis

The microemulsions morphology was studied by atomic force microscopy (AFM) (JPK, Nano Wizard 2, and Berlin Germany). For AFM analysis, samples were diluted by deionized water and then the clean mica surface were smeared by a drop of the diluted sample, followed by being lyophilized to prepare a fixed sample. All AFM images were processed and analyzed using the JPK Data Processing software.

#### 2.4.4 Differential scanning calorimetry (DSC)

Differential scanning calorimetry (DSC) of Analysis (Mettler Toledo, model Star SW 9.30, Schwerzenbach, Switzerland) was applied for thermal analysis of the microemulsions BSA-PEG NPs Each sample was put inside the sealed aluminum-lead pans and run at a scanning rate of 15 °C.min^−1^ from 0 °C to 300 °C.

#### 2.4.5 Conductivity measurements

The electrical conductivity of the prepared MEs samples were measured by conductivity meter Metrohm Model 712. This experiment was performed at room temperature (25 ± 1 °C). All measurements were repeated three times.

#### 2.4.6 pH determination of ME

After calibration of pH meter with buffer solution of pH 4.0, 7.0, and 9.0, the pH values of MEs samples were measured at 25 °C by a Corning 220 pH meter (Cole-Palmer, Teddington, UK). This experiment aimed to investigate the influence of ME type and presence or absence of BSA NP on pH and compare the results with those of blood plasma. The measurement of pH of MEs samples were done in triplicate and the mean values were calculated [14].

#### 2.4.7 Refractive index of ME

Refractive index for MEs samples was measured by refractometer M46.17/63707 (Higler and Walts Ltd., England, UK) at 25 °C by pouring one drop of solution in the slide.

#### 2.4.8 Limpidity test (percent transmittance)

Transparency of the prepared MEs samples was determined by measuring the amount of transmittance using spectrophotometry (Shimadzu, UV-160, Japan) [15]. Transmittance of ME was measured at 633 nm with double distilled water taken as blank. It was done three times for each sample [13].

#### 2.4.9 Rheological behavior

The rheological properties of microemulsion and microemulsion hybridized with BSA NPs samples were measured using a viscometer (Brookfield Viscometer LTD) [16]. In all of the experiments, 800 μL of ME was placed into the surface of the reading plate, and the excess sample was removed. The data were collected using Brookfield Viscometer LTD Software.

### 2.5 Stability of ME

To confirm the physical stability of the prepared MEs, the particle size distribution was investigated every 15 days for 90 days at room temperature by DLS.

### 2.6 Cell culture and cell viability

For preliminary study of cell viability of the prepared MEs, the MTT (3-[4, 5-dimethylthiazol-2-yl]-2, 5-diphenyl tetrazolium bromide) assay test was selected. HFF-2 and HEK-293 cells were cultured in Dulbecco's modified eagle medium (DMEM) with 10% Penicillin-Streptomycin (10% Pen-Strep) and 10% heat-inactivated fetal bovine serum (FBS) in an incubator at 37 °C under an atmosphere of 5% CO_2_ and 80% relative humidity. HFF-2 and HEK-293 cells were seeded at the density of 6×10^3^ cell per well in 96-well plates for 48 h (80% confluency). Then the cells were incubated for 48h with 200 μL of fresh culture medium containing six various concentrations ranging from 0-50 μg/mL of T-ME and the same serial concentration of T-BSA-ME and some wells without treatment as control. After the incubation time, the culture medium was removed and washed with PBS. Then, 20 μL of MTT (4 mg/mL) solution was added to each well and the plates were incubated in a humid environment containing 5% CO_2_ at 37 °C. After 4 h of incubation, the medium was removed, and then 100 μL of dimethyl sulfoxide (DMSO) was added to each well for dissolving the violet formazan crystals. After shaking the plates for 15 min, cell viability was quantified by measuring the absorbance of the formazan at the wavelength of 570 nm with a microplate reader (Spectra Max 190). The data were expressed as the percentage of viable cells compared to the survival of the control group (untreated cells as controls of 100% viability). The data were expressed as the mean ± standard deviation (SD) of three individual measurements.

### 2.7 Hemolysis assay

Hemolysis refers to the damage of erythrocytes membrane, leading to the leaking out of erythrocyte intracellular content (Hemoglobin) into blood plasma. The occurrence of hemolysis inside the body can lead to anemia, jaundice and other pathological conditions, which may become life-threatening. Hemoglobin is one of the components of the red blood cells, carrying efficiently oxygen from lungs to the tissues of the body. It also plays an important role in transportation of carbon dioxide and hydrogen ions back to the lungs. However, extracellular hemoglobin is toxic and may affect vascular, myocardial, renal and central nervous system tissues. Since the microemulsion hybridized with BSA NPs is to be used for biomedical applications, hemolysis has to be addressed as an important issue in this regard. The toxicity degree (hemolytic effect) of MEs samples was evaluated by a previously reported method [17]. Healthy human red blood cells (RBC) were taken from the blood bank of Mousavi Hospital (Zanjan, Iran), collected in tubes with heparin and centrifuged at 4000 rpm for 5 minutes. The plasma was removed, and RBCs were washed three times with PBS. The purified RBC was re-suspended in isotonic PBS until it was diluted to 10% of their initial concentrations, i.e. six different mass concentrations of T-ME and T-BSA-ME (10, 40, 70, 100, 130 and 160 μg/ml). The %1 Sodium dodecyl sulfate (SDS) and 500μl PBS was used as positive control (100% Hemolysis) and negative control (0% hemolysis), respectively. Aliquot of 200 μl RBC were added to the tubs. Then, all samples (sample treatment, positive control and negative control) were added to different tubs and incubated for 4 h in a 37 °C water bath. After incubation, the tubes were centrifuged for 10 min at 4000 g at room temperature. Finally, 200 μl of the hemoglobin released into the medium was transferred to a 96-well plate. The absorbance was measured at 541 nm using a plate reader (Eppendorf Bio Photometer). This test was repeated three times. The amount of hemolysis in percent was calculated as follows:

$$\text{Hemolysis}\left(\%\right)=\frac{A_{sample}-A_{negative}}{A_{positive}-A_{negative}}\times 100$$

where A_sample_ was represents mean absorbance of ME (test). Likewise, A_negative_, and A_positive_ represent mean absorbance of negative and positive control, respectively.

### 2.8 LD_50_ assay

Different chemicals, such as triacetin, Tween 80, Span 80, BSA, and PEG that are used for ME formulations have different toxic effects. LD_50_ is one of the methods of measuring the short-term toxic potency (acute toxicity) of a prepared ME. In order to study the safety of the prepared ME, an acute oral toxicity study was done in adult mic. 10 Adult mice of an average weight of 30–35 g were taken in an ideal laboratory condition as per OECD [18]. To carry out LD50 assay, T-ME and T-BSA-ME in concentrations ranging from 175 to 5000 mg/kg were orally administered to each animal. All Animals were weighed before administration and also, after one day and one week. Likewise, during the test, any physical activities and behavioral changes of animals were monitored. If all the animals survived after 24 h, two other animals were treated at the highest does. If these animals survived too, the LD50 would be more than the dose limit and the test was stopped.

### 2.9 Leukocyte proliferation assay

MEs have been used in many areas including anticancer drug delivery, local drug delivery, vaccine delivery, clinics. Hence, investigation of the effects of MEs on lymphocyte activation is the most important issue. For this assay, in order to isolate lymphocytes, 4 mL of blood/PBS mixture were poured in the conical centrifuge tube and then, 3 mL of Ficoll-Paque solution was slowly added and then, centrifuged for 30 min at 900 x g, 19 °C, without brake. After the removal of supernatant, the remaining solution (mononuclear cell layer) was transferred into another centrifuge tube and cells ware washed by adding an excess of HBSS (Hank's balanced salt solution), and centrifuged for 10 min at 400 x g, 19 °C. In the next step, mononuclear cells were cultured in RPMI-1640 (Roswell Park Memorial Institute medium-1640) medium in an incubator at 37 °C under an atmosphere of 5% CO_2_ and 80% relative humidity. Monoclonal cells were seeded at the density of 2.5×10^6^ cell per well in 96-well plates for 48 h. Then, the cells were incubated for 48h with fresh culture medium, making them ready for evaluation of the lymphocyte proliferation activity (or inhibition). Lymphocyte proliferation activity was described with various concentrations of T-ME or T-BSA-ME, including 2.0. 0.2, 0.04, and 0.008 mg/ml. Also, some wells without treatment and some wells with 50 μl of 20 μg/ml of PHA-P (phytohemagglutinin) were used as negative and positive control, respectively. Likewise, lymphocyte proliferation inhibition was evaluated with various concentrations of T-ME or T-BSA-ME, including 4, 0.4, 0.08, and 0.016 mg/ml, mixed with 50 μl of 20 μg/ml PHA-P. After being incubated for 48 h, the culture medium was removed and washed with PBS. Then, 20 μL of MTT (4 mg/mL) solution was added to each well and the plates were incubated in a humid environment containing 5% CO_2_ at 37 °C. After 4 h of incubation, the medium was removed, and then, 100 μL of dimethyl sulfoxide (DMSO) was added to each well for dissolving the violet formazan crystals. After shaking the plates for 15 min, cell viability was quantified by measuring the absorbance of the formazan at the wavelength of 540 nm with a microplate reader. The data were expressed as the percentage of proliferation and proliferation inhibition that are calculated as follows:

$$\%\mathrm{proliferation}=\frac{\mathrm{Mean}\;OD_{test\;sample}-\mathrm{Mean}\;OD_{untreated\;cells}}{\mathrm{Mean}\;OD_{untreated\;cells}}\times 100$$

$$\%\mathrm{proliferation}\;\mathrm{inhibition}=\frac{\mathrm{Mean}\;OD_{Positive\;control}-\mathrm{Mean}\;OD_{Positive\;control+ME}}{\mathrm{Mean}\;OD_{Positive\;control}-\mathrm{Mean}\;OD_{untreatedcells}}\times 100$$

where OD_untreated cell_ represent optical density of plates without test sample (as negative control). Likewise, OD_test sample_, OD_positive control_, and OD_positive control+ME_ were represents, optical density of plates in presence of MEs (test samples), PHA-P (as positive control), and PHA-P with ME, respectively.

### 2.10 Statistical analysis

Statistical analysis of the data was carried up by the *Graph Pad Prism* 7 software. The experiments were repeated at least three times, and the data were expressed as means ±SD. For normally distributed data, one-way ANOVA with repeated measures was used to compare within the same group and Tukey's test for correction. Likewise, one-way ANOVA with Dunnett's test was used to compare mean between different groups. For the non-normally distributed data, the Kruskal-Wallis test and Fisher‘s exact test were employed for correction and categorical variables, respectively. P value <0.05 and ns considered as significant and not significant difference, respectively.

## 3 Results and discussion

### 3.1 Optimum formulation of T-ME

**Fig. 1:**
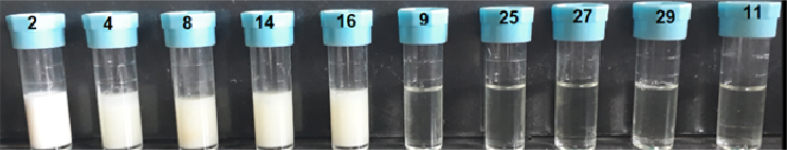
Study of microemulsion system stability in various *component percentages.*

In order to fabricate ME systems, two phases were really needed: the aqueous phase that includes water, surfactant, and co-surfactant; the oil phase that includes oil composition. Propylene glycol (PG) and glycerol were selected as co-surfactants for screening. Martino A. and Lwanaga T showed that PG and glycerol incorporated into the surfactant layer, leading to an increase in the interfacial fluidity, due to the great solubility of PG and glycerol in water which in turn can decrease the polarity of the water. Hence, PG and glycerol could increase the flexibility of surfactant, allowing water droplets to merge and incorporate more water. As a result, PG and glycerol created a large single-phase region [9, 19]. Tween 80 was selected as surfactant due to its low toxicity, biocompatibility and high HLB value (HLB = 15). Therefore, it is suitable for O/W type of ME [20]. Furthermore, Span 80 (HLB = 4.3) and Tween 80 were mixed as surfactant, characterized by a viscous, lipophilic, emulsifying liquid agent and low HLB value. The results showed that the mixture of Tween 80 and Span 80 surfactants with significant different HLB values produce stable formulations (Table 1 and Fig. 1). Span 80 with low HLB value and Tween 80 with high HLB value were dissolved in oil phase and water, respectively, enabling them function together and leading to the stability of formulation [9]. Therefore, in ME composition, surfactant and co-surfactant type, as well as their concentrations are main parameters.

**Table 1:** Composition and evaluation of triacetin MEs formulations.

Formulation	Triacetin	Tween 80 (%W/W)	Span 80 (%W/W)	PG (%W/W)	Glycerol (%W/W)	Water (%W/W)	State
1	8.33	20.83	-	4.16	-	66.68	Separation phase
2	8.33	20.83	-	-	4.16	66.68	Separation phase
3	6.78	21.18	-	4.23	-	67.81	Separation phase
4	6.78	21.18	-	-	4.23	67.81	Separation phase
5	5.17	21.55	-	4.31	-	68.97	Separation phase
6	5.17	21.55	-	-	4.31	68.97	Separation phase
7	8.44	16.86	2.87	4.22	-	67.61	Slightly Cloudy- separation phases after week
8	8.44	16.86	2.87	-	4.22	67.61	Slightly Cloudy- separation phases after 10 days
9	6.87	17.18	2.92	4.29	-	68.74	Slightly Cloudy and stable
10	6.87	17.18	2.92	-	4.29	68.74	Slightly Cloudy and stable
11	**5.24**	**17.48**	**2.97**	**4.37**	**-**	**69.94**	**Transparent**
12	5.24	17.48	2.97	-	4.37	69.94	Slightly Cloudy and stable
13	8.64	14.69	3.19	4.32	-	69.16	Cloudy
14	8.64	14.69	3.19	-	4.32	69.16	Cloudy
15	7.05	14.95	3.25	4.39	-	70.36	Cloudy
16	7.05	14.95	3.25	-	4.39	70.36	Cloudy
17	5.39	15.21	3.31	4.47	-	71.62	Cloudy
18	5.39	15.21	3.31	-	4.47	71.62	Cloudy
19	8.42	18.53	1.43	4.23	-	67.39	Slightly Cloudy- separation phases after 2 week
20	8.42	18.53	1.43	-	4.23	67.39	Slightly Cloudy- separation phases after 2 week
21	6.86	18.85	1.46	4.28	-	68.55	Slightly Cloudy and stable
22	6.86	18.85	1.46	-	4.28	68.55	Slightly Cloudy- separation phases after 8 days
23	5.23	19.18	1.48	4.36	-	69.75	Slightly Cloudy- separation phases after 12 days
24	5.23	19.18	1.48	-	4.36	69.75	Slightly Cloudy- separation phases after 10 days
25	8.31	18.27	2.82	4.16	-	66.44	Transparent
26	8.31	18.27	2.82	-	4.16	66.44	Transparent
27	6.76	18.58	2.87	4.22	-	67.57	Transparent
28	6.76	18.58	2.87	-	4.22	67.57	Transparent
29	5.15	18.90	2.93	4.29	-	68.73	Transparent
30	5.15	18.90	2.93	-	4.29	68.73	Transparent

Hence, in order to obtain the best formulation of O/W ME, different ratio of triacetin, water, surfactant, and co-surfactant were evaluated (Table 1). In the first six formulations, MEs were prepared without Span 80. The results showed that aqueous phase and organic phase were separated. The results indicated that Span 80 had an important role in the stability of formulation, which was confirmed with some previous study [21, 22]. In six formulation (13–18 formulations), although Span 80 was mixed with Tween 80 as surfactant, the prepared MEs were cloudy in appearance. This may resulted from the insufficiency of surfactant mixture (Tween 80 & Span 80) needed for the fabrication of microemulsion. With an increase in the surfactant ratio in the range of 19.73%–20.66%, microemulsions showed slightly cloudy states. The reason was that the ratio of Span 80 was low and not sufficient for ME stability [23, 24]. Some of the prepared microemulsions (11 and 25 to 30 formulations) showed transparent states. The results indicated that in addition to the surfactant ratio, co-surfactant type and co-surfactant amount also played an important role in the stability and Physical appearance of formulations. Among all transparent formulations, 11 formulations were selected for further tests in this paper, since they had less surfactant. Another reason for such a selection was the toxicity of surfactant in high concentration. Furthermore, this was very important in terms of application and economy.

### 3.2 Characterization of particle size and morphology

**Fig. 2:**
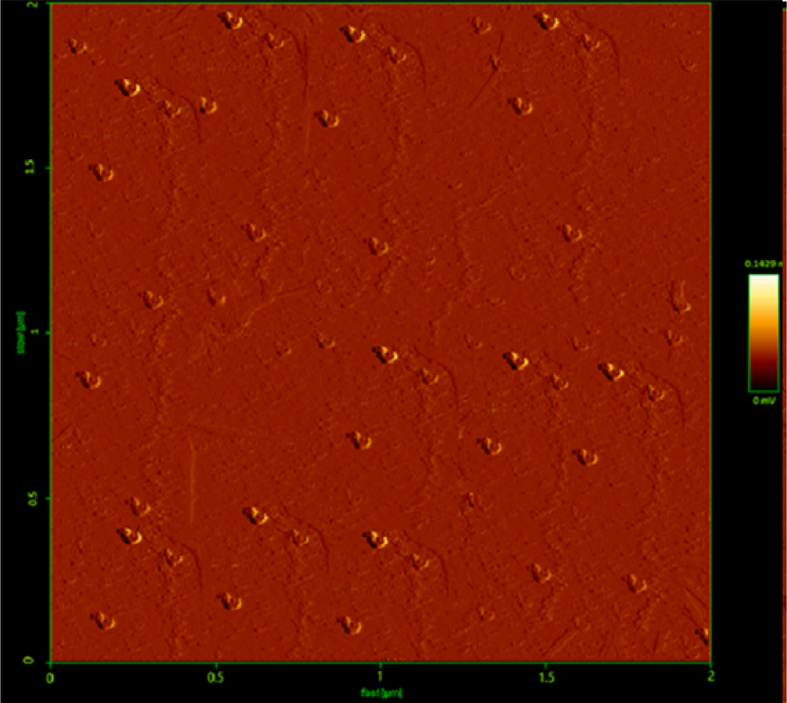
Atomic force microscopy (AFM) image of T-BSA-ME.

The formation of T-ME hybridized with BSA-NPs (T-BSA-ME) was approved by AFM. In detail, T-BSA-ME showed a globular shape and homogeneous spherical morphology, as expected (Fig. 2). Also, the size of T-ME and T- BSA-ME-were evaluated by dynamic light scattering system (DLS).

**Fig. 3:**
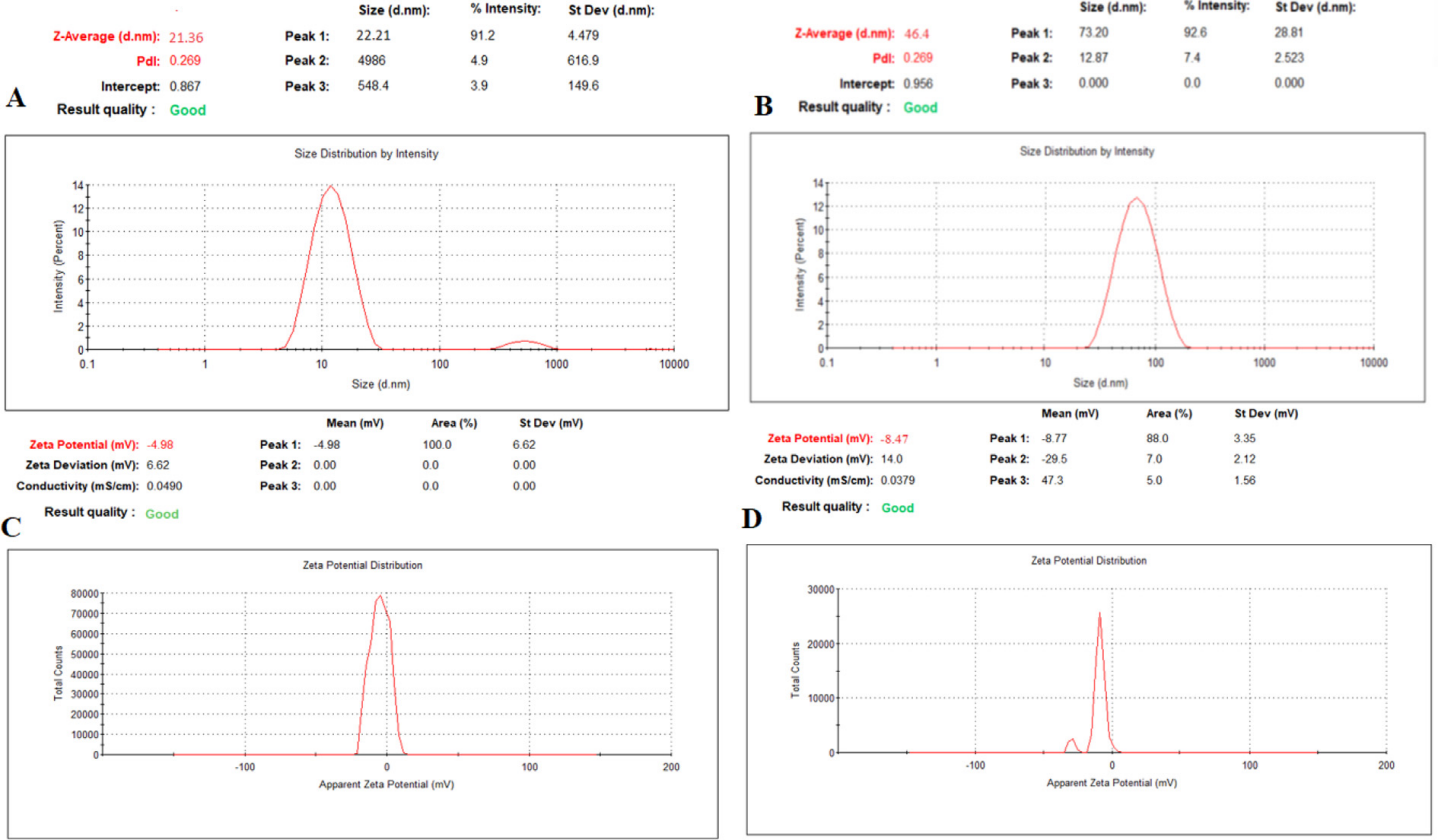
Size distribution by intensity of A) T-ME, B) T-BSA-ME and ζ-potential of C) T-ME, D) T-BSA-ME.

As can be seen in Fig. 3, the average size of T-ME and T- BSA-ME-were found to be around 21.36 nm and 46.4 nm, with ζ-potential of -4.98 and -8.47 mV, respectively. The T-BSA-ME size was higher than that of T-ME because of the presence of BSA NPs, The negative charge value of ζ-potential was resulted from hydroxyl groups of surfactants, co-surfactant, and BSA NPs which contain amine and several functional groups with high electronegativity such as hydroxyl, amid and carboxyl functional groups [25]. Hence, the ζ-potential of T-BSA-ME was higher than that of T-ME [26].

### 3.3 FT-IR analysis

**Fig. 4:**
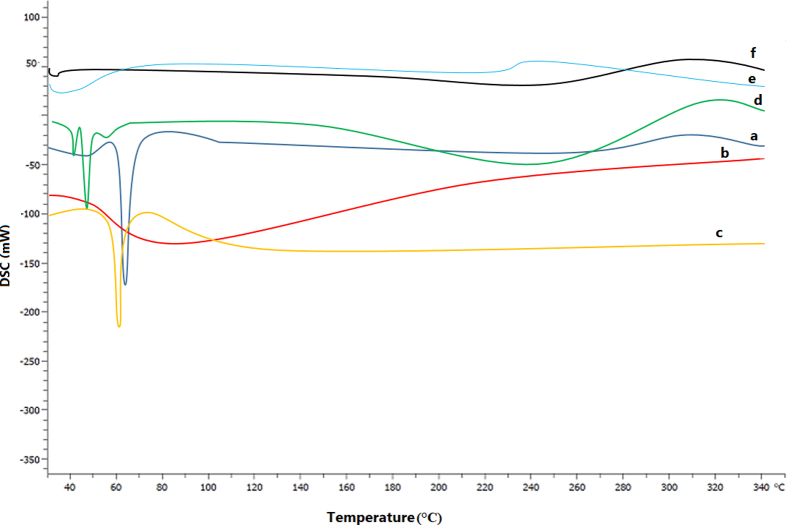
FT-IR spectra of (a) T-ME, (b) BSA NPs, (c) T-BSA-ME.

FT-IR spectroscopy was carried out to confirm the components of the T-ME hybridized with BSA NPs. As can be observed in Fig. 4, the FTIR spectrum analysis was used to indicate the compositions of BSA NPs, T-ME, and T-BSA-ME. The characteristic absorption peaks of BSA at 1700 and 1500 cm−1were attributed to C=O stretching vibrations (amide-I) and N–H bending vibrations (amide-II), respectively [27, 28]. All functional groups in T-ME (Fig. 3a) were similar to those of BSA NPs (Fig. 3b). As can be seen in Fig. 4, all the characteristic absorption peaks of T-ME (Fig. 4a) and BSA NPs (Fig. 4b) could be observed in the FTIR spectra of T-BSA-ME. Thereby, the results demonstrated that BSA NPs was successfully hybridized with T-ME.

### 3.4 Differential scanning calorimetry (DSC)

**Fig. 5:**
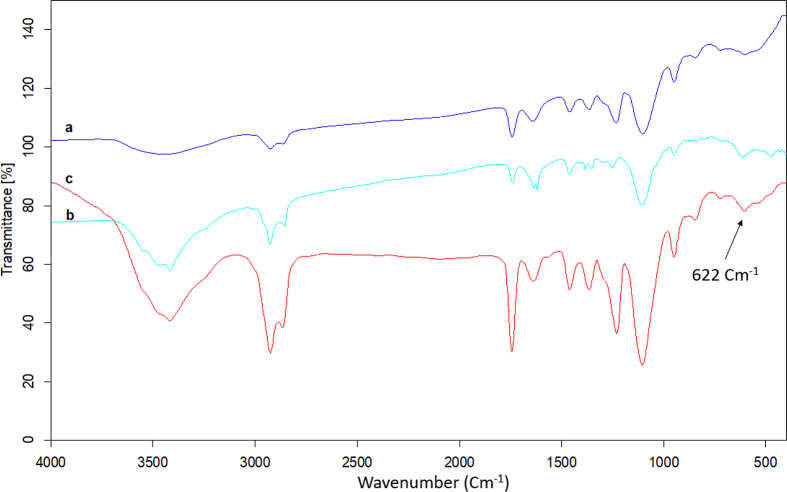
DSC thermograms of BSA NPs (a), triacetin (b), T-ME (c), and T-BSA-ME (d) in the temperature range of 30–340 °C.

DSC thermogram of BSA NPs, triacetin, T-ME, and T-BSA-ME was performed to evaluate each sample's thermal behavior (Fig. 5). Triacetin was characterized by the existence of an endothermic peak at 51.65 °C and BSA NPs, about 78.52 °C, while the T-ME and T-BSA-ME systems had no peak visible near the triacetin and BSA NPs's melting point. As can be observed, triacetin and BSA NPs were amorphous in ME systems.

### 3.5 Rheological behavior

**Fig. 6:**
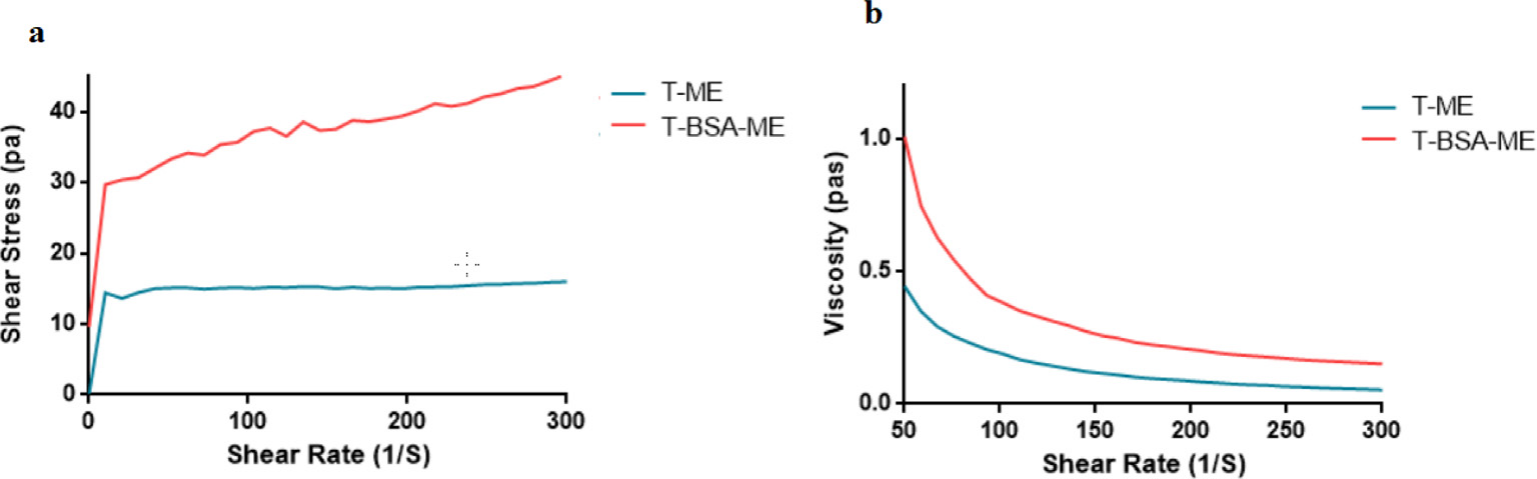
Rheological behavior of T-ME and T-BSA-ME.

Rheological analysis of MEs is very important for many reasons including investigation of structural properties, system stability, handling, storage, and pipeline transportation of MEs [29, 30]. Fig. 6 shows the shear stress and viscosity as a function of the shear rate for the T-ME and T-BSA-ME, at room temperature and at atmospheric pressure. Fig. 6a revealed that, at lower shear rates (˂50 S^−1^), the shear stress increased urgently, shear stress for T-ME increased slowly, while in T-BSA-ME, it was approximately constant. To describe the rheology behaviors, and to compare the candidate models for two MEs (T-ME & T-BSA-ME) datasets, Akaike's An Information Criterion (AIC) function were calculated for each model to help determine which model is the best.

The AIC is defined as follows [31]:

$$\text{AIC}=2\times P+N\;\log\frac{RSS}{N}$$

where P, N, and RSS represents, number of parameters in the fitted model, the number of data set and residual sum of squares (RSS), respectively. A good model is the one that has minimum AIC among all the other models [32]. As can be shown in Table 2, the data obtained from all candidate models confirmed that both microemulsion systems (T-ME & T-BSA-ME) exhibited a non- Newtonian viscous behavior.

**Table 2:** Description of different models for MEs systems.

Model	Equation	P	T-BSA-ME				T-ME			
			AIC	n	τ_0_	*K*	AIC	n	τ_0_	*K*
Newtonian	τ = kƴ	1	**72.14**			0.20	**52.55**			0.08
Herschel-Bulkley	τ = τ_0_ + kƴ^n^	3	**6.08**	0.20	9.91	10.83	**-30.28**	0.01	0	8.14
Bingham	τ = τ_0_ + kƴ	2	**37.8**		27.89	0.06	**27.78**		12.67	0.01
Power-Law/Ostwald	τ = kƴ^n^	2	**22.79**	0.14		19.31	**-32.28**	0.03		13.14

τ: Shear Stress, τ_0_: Yield Stress, ƴ: shear Rate, *K:* Consistency Index, n: Flow Index, P: Number of Parameters.

Hence, according to the AIC value, the T-ME and T-BSA-ME systems were characterized by Power-Law/Ostwald and Herschel-Bulkley models, respectively. Evidently, the results confirmed that MEs systems (T-ME & T-BSA-ME) exhibited shear thinning pseudoplastic behavior because ??-values obtained were less than unity (??<1). Moreover, the hybridization of BSA NPs with T-ME did not alter the flow behavior of the MEs. Shear thinning behavior may be due to phase segregation in MEs systems. Furthermore, ??-values decreased with the hybridization of BSA NPs in MEs system, revealing an increasing trend in the shear thinning characteristic of the suggested system. As can be seen in Table 2, the consistency index (??) increased with the hybridization of BSA NPs in MEs system, increasing the apparent viscosity of the T-BSA-ME that may be due to different formation and more organized microstructure. The viscous flow behavior of T-ME and T-BSA-ME were related to the apparent yield stress resulting from interactions between BSA NPs and the continuous or external aqueous phase [33, 34]. Meanwhile, the interactions between the BSA NPs surface and the aqueous phase might increase the stiffness, as pointed out by Zhong [35] and Hato [36]. Thus, the hybridization of BSA NPs with T-ME leads to an increase in viscosity. Aqueous media (continuous phase) as a polar solvent in the presence of BSA NPs resulted in the bulk viscosity and creation of a new internal phase. As a consequence, the presence of an internal aqueous phase increased dispersion viscosity, particularly in low shear rate range, as shown in Fig. 6b. Nonetheless, at high shear rates, the differences were significantly dampened.

### 3.6 Physicochemical characterizations

The physicochemical characterizations of the MEs were shown in Table 3. The conductivity was profoundly sensitive both to temperature and composition. The composition of O/W ME system was fabricated with triacetin, Tween 80, Span 80, PG and water.

**Table 3:** Physicochemical characterization of T-ME and T-BSA-ME.

Formulations	Conductivity (ms/cm)	pH	RI	%Transmittance
T-ME	2.49	5.23	1.38	93.9
T-BSA-ME	1.47	6.16	1. 21	57.32

**Fig. 7:**
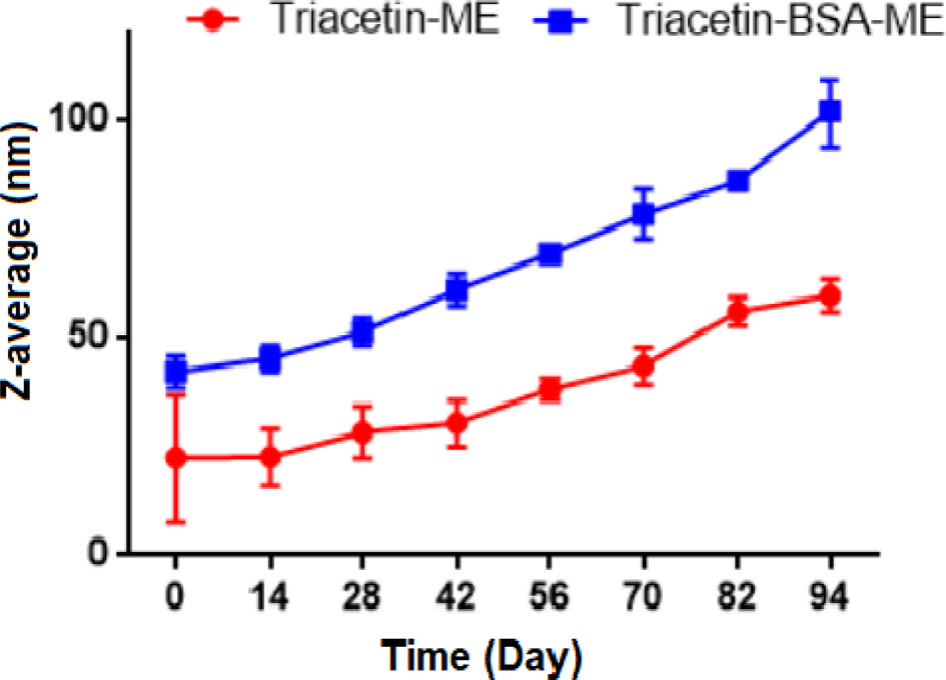
Stability curves of T-ME and T-BSA-ME.

In the case of the O/W MEs systems when the content of water is over 65 percent as the continuous phase, the conductivity of the systems increased. However, the W/O (water-in-oil) systems had low conductivity. The electrical conductivity was used to determine the type of microemulsions [37]. The electrical conductivity coefficient of T-ME and T-BSA-ME was revealed in 1.47 ms/cm and 2.49 ms/cm, respectively. the high value of conductivity coefficient is characteristic of oil-in-water (O/W) type of MEs (Table 3) [38, 39]. As can be seen, the conductivity coefficient of T-ME was higher than that of T-BSA-ME which was related to the higher percentage of water in T-ME compared with that of T-BSA-ME. The refractive index (RI) of T-ME and T-BSA-ME was found to be around 1.38 and 1.2, respectively. Therefore, as we discussed before, the RI of the system became equal to the water's RI (1.33). Furthermore, the transmittance of T-ME and T-BSA-ME was found to be 93.9% and 57.32%, respectively, indicating that the prepared ME were a transparent, single phase liquid, while the transmittance of T-BSA-ME was lower than that of T-ME that could be related to the presence of BSA NPs in the formulation. The pH value of T-ME and T-BSA-ME was in the range of 5.23–6.16. The pH value of the T-BSA-ME was less than that of T-ME, which could be associated to the presence of acidic groups at BSA structure. The MEs samples (T-ME & T-BSA-ME) stability was approved physically. MEs systems were also of good thermodynamic stability, due to ultralow interfacial tension between the oil and water phases. In order to describe the ME stability, the particle structure integrity and the possibility of aggregation were evaluated. Therefore, the particle size of ME as a function of incubation time was monitored for 3 months. As shown in Fig. 7, Z-average of T-ME and T-BSA-ME did not increase significantly throughout the measurement period.

### 3.7 Cytotoxicity study

**Fig. 8:**
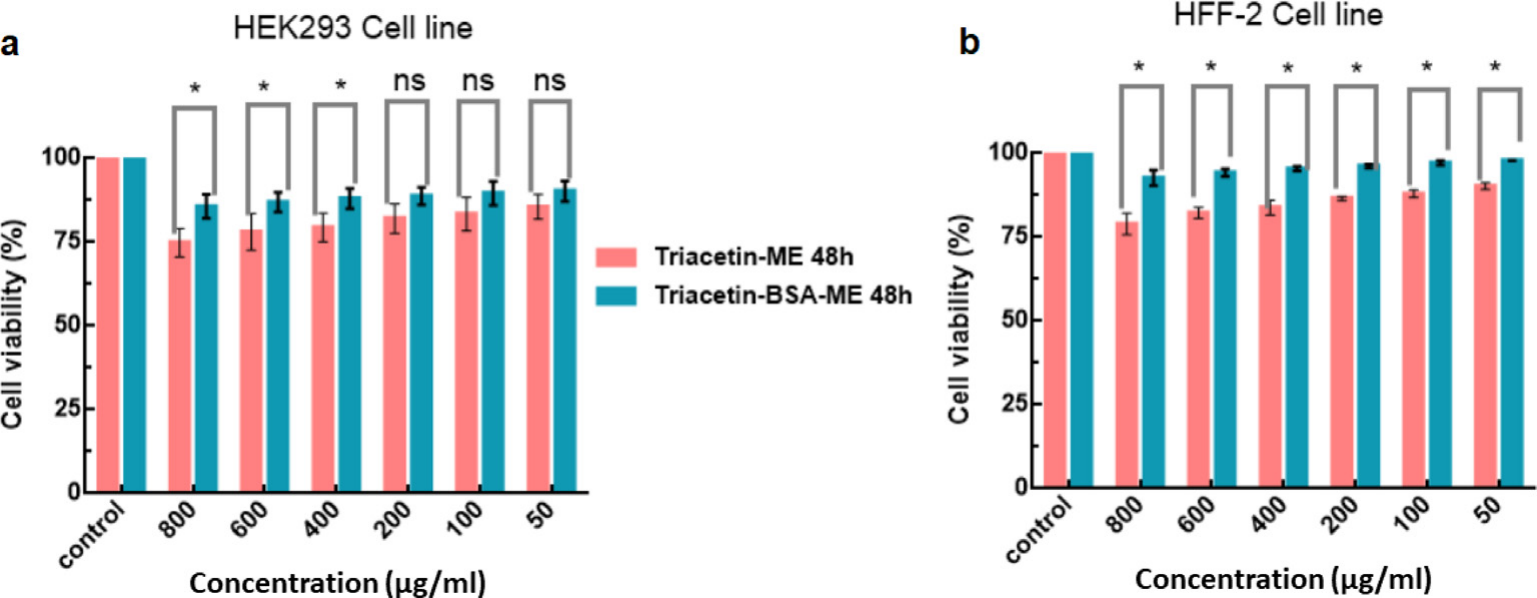
The cell viabilities of the T-ME and T-BSA-ME on HEK-293 cell line (a), and HFF-2 cell line (b). Data are represented as mean ± standard deviation (n = 3) *p < 0.05 and ns considered as significant difference and not significant, respectively.

For clinical and biomedicine applications, toxicity is a critical factor that should be considered. Biomedicine applications of MEs are intentionally designed for several clinical uses. It is essential to approve that this offer does not have any adverse effect. Besides T-ME, BSA NPs hybridized with T-ME should also be evaluated in terms of the cytotoxicity effect on both HFF-2 and HEK-293 cell lines. The cytotoxicity effect of T-BSA-ME on both HFF-2 and HEK-293 cell lines were evaluated and measured by MTT assay. The cells were incubated with various concentrations (50, 100, 200, 400, 600, 800 μg/ml) for 48 h in a 5 % CO_2_ atmosphere. As can be seen, Fig. 8 compares the cells viability in different T-ME and T-BSA-ME concentrations.

The cell viabilities of HFF-2 cells after being treated with various concentrations of T-ME and T-BSA-ME ranged from 78.99 ± 3.18 to 90.31 ± 1.01% and 92.71 ± 5.53 to 97.9 ± 0.02% after 48 h, respectively. At the same time, viability of HEK-293 cells, after treatment with various concentrations of T-ME and T-BSA-ME, were obtained to be in the range of 74.84 ± 4.2 and 85.56 ± 3.6% and 85.75 ± 3.52 and 90.28 ± 3.04% after 48 h, respectively. Thereby, T-ME and T-BSA-ME were not significantly toxic on HFF-2 and HEK-293 cell lines, suggesting that MEs systems (T-BSA-ME especially) were of high-level biocompatibility and likely to be used for further in vivo applications. Data were analyzed using ANOVA, *p < 0.05 and ns considered as significant and not significant difference, respectively.

### 3.8 Hemolysis assay

**Fig. 9:**
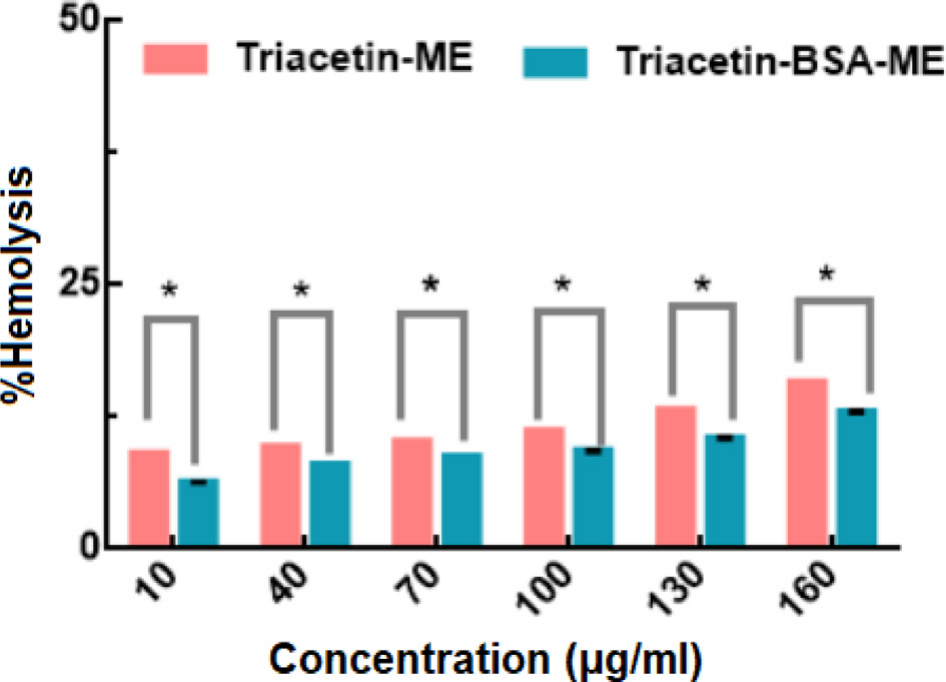
Percentage of hemolysis induced by T-ME and T-BSA-ME at various concentration and 37 °C, conditions. Data are represented as mean ± standard deviation (n = 3) *p < 0.05 considered as significant difference.

For biomedicine uses, MEs systems enter the body and interact with tissues and cells directly. It is necessary for the evaluation of their biocompatibility [40]. To determine the biocompatibility of the T-ME and T-BSA-ME, the MEs systems must have a blood compatibility with minimal hemolytic effect on human red blood cells. As can be seen in Fig. 9, the blood compatibility of T-ME and T-BSA-ME were evaluated at 10–160 μg/ml concentration range. The hemolytic activity of the samples was quantitatively determined by the evaluation of the supernatant absorbance at 540 nm (hemoglobin) with Eppendorf Bio Photometer [17]. The hemolytic activity of T-ME and T-BSA-ME in various mass concentrations was in the range of 6.32 ± 0.02 to 13.00 ± 0.12% and 9.09 ± 0.00 to 15.87 ± 0.00%, respectively. The hemolytic activity of MEs systems may be related to the presence of the surfactant that, when increases in the sample, causes an increase in the hemolytic rate [41]. Likewise, the T-ME revealed significantly higher hemolytic rates compared to T-BSA-ME in similar concentration that was due to the surfactant used. Data were analyzed using ANOVA, *p < 0.05 was considered as significant difference.

### 3.9 LD50 and acute toxicity

For Median Lethal Dose (LD 50) test, we treated mice with a series of doses. Mice were treated with T-ME and T-BSA-ME, ranging from 17.5 to 5000 mg/kg. To find of out maximum safe dose, we used a control group administrated with saline and treatment groups were administrated with 17.5, 175, 1750, and 5000 mg/kg of T-ME and T-BSA-ME. The changes in the mice weight after 24 h and 7 days were around 1.58 ± 3.64% and 5.42 ± 1.78%. During the treatment, none of the mice died. So, using OCED and Hodge and Sterner scale, we concluded that the MEs systems introduced were practically none toxic [42].

### 3.10 Leukocyte proliferation assay

Lymphocyte proliferation is one of the basic characteristic of the response of lymphocytes to antigenic stimulation. In order to measure the ability of ME system to induce a proliferative response in human Lymphocyte, along with proliferation percentage, the percentage of proliferation inhibition should be evaluated. For this Assay, Lymphocyte were isolated from human whole blood using Ficoll-Paque solution and then, it was cultured. The amount of Proliferation showed MEs ability to induce leukocytes proliferation compared with untreated sample. The amount of proliferation inhibition also showed MEs inhibitory effects on PHA as a proliferative factor [43, 44].

**Fig. 10:**
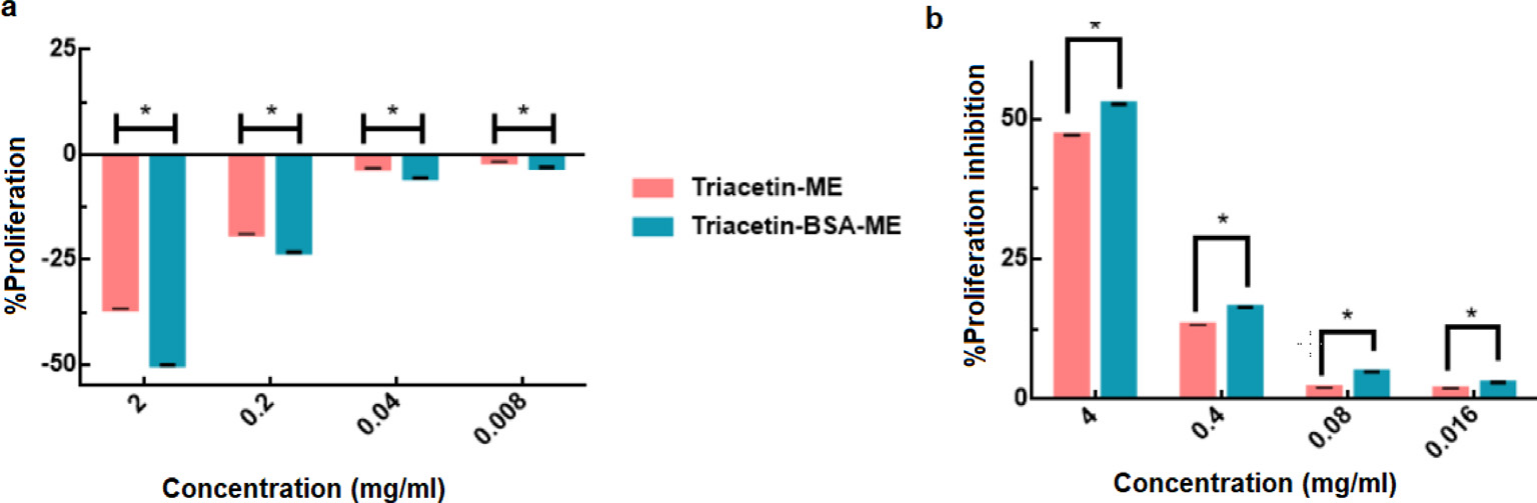
The proliferation (a) and proliferation inhibition (b) rate of T-ME and T-BSA-ME. Data are represented as mean ± standard deviation (n = 5) *p < 0.05 considered as significant difference.

As can be seen in Fig. 10, the proliferation rate of triacetin and T-BSA-ME in various concentrations ranged from-36.6 to -1.61% and -49.98 to -2.95%, respectively. These results revealed that the proliferation rate of the suggested MEs systems was lower than that of untreated samples (especially in high concentration). Likewise, the proliferation inhibition rate of triacetin and T-BSA-ME in various concentrations ranged from 47.15 to 1.93% and 52.7 to 2.99%, respectively. The results indicated that the suggested MEs systems could act as a Lymphocyte proliferation inhibitory in presence of PHA as a proliferative factor (positive control). Data were analyzed using ANOVA, and*p < 0.05 was regarded as significant difference.

## 4 Conclusion

The present paper aimed to design and prepare T-BSA-ME, practically none toxic with potential applications such as co-delivery and multifunctional systems. In vitro and in vivo cytotoxicity evaluation revealed that the suggested MEs systems were safe and none toxic. Likewise, they were biocompatible, not active for Leukocyte Proliferation. The non-toxicity of the systems significantly increased their applicability in the development of in-vitro and in-vivo biomedicine fields such as brain co-delivery or multifunctional system in neurodegenerative and cancer diseases. It is worth noting that this study established the foundation for effective preparation of T-BSA-ME for further in vitro and in vivo studies (in progress) on conjugates of T-BSA-ME with drugs, drug candidates, targeting agent, and molecular probes.

## Declarations

### Author contribution statement

Mahmoud Gharbavi: Performed the experiments; Analyzed and interpreted the data.

Hamidreza Kheiri Manjili: Conceived and designed the experiments.

Jafar Amani: Contributed reagents, materials, analysis tools or data.

Ali Sharafi: Conceived and designed the experiments; Performed the experiments.

Hossein Danafar: Conceived and designed the experiments; Wrote the paper.

### Funding statement

This research did not receive any specific grant from funding agencies in the public, commercial, or not-for-profit sectors.

### Competing interest statement

The authors declare no conflict of interest.

### Additional information

No additional information is available for this paper.
